# A thematic-cognitive perspective for exploring the writing skills of children: a textual analysis using ENA

**DOI:** 10.3389/fpsyg.2025.1494111

**Published:** 2025-08-11

**Authors:** Jianheng Zhang, Tiong-Thye Goh, Dexin Chen, Yuan Gong, Bing Yang, Liqin Pan, Ting Song, Shiqi Yu, Hanzhen Li

**Affiliations:** ^1^School of Computer Science, Hubei University, Wuhan, China; ^2^School of Information Management, Victoria University of Wellington, Wellington, New Zealand; ^3^Wuhan Changqingshu Experimental School, Wuhan, China; ^4^School of Foreign Language, Hubei University, Wuhan, China; ^5^School of Education, Hubei University, Wuhan, China; ^6^School of History and Culture, Hubei University, Wuhan, China

**Keywords:** writing ability, epistemic network analysis, writing education, children, writing topic

## Abstract

**Introduction:**

Primary school is a critical period for children’s language development, coinciding with rapid cognitive growth that supports the emergence of writing skills. Understanding how children’s cognitive structures manifest in writing is essential for improving instructional strategies.

**Methods:**

This study employed epistemic network analysis (ENA) to encode and analyze six years of student writing data. Cognitive network maps were constructed to examine developmental trends and differences across grades and genders from both subject-matter and cognitive perspectives.

**Results:**

The analysis demonstrates ENA’s effectiveness in visualizing the cognitive features embedded in written texts. Distinct patterns emerged across subjects, grades, and genders, revealing a complex and nuanced cognitive network structure.

**Discussion:**

These findings highlight important nuances in children’s writing development. Recognizing subject-specific, developmental, and gender-related cognitive differences can inform more personalized and effective writing instruction.

## 1 Introduction

Writing is not only a means for humans to express their desires and innermost feelings, but also a crucial skill in various settings, including social interactions, workplace tasks, and academic research (Wise, 2005). It plays a pivotal role in the advancement of scientific, social, and mathematical understanding (Graham et al., 2020). However, a significant issue in primary education is the limited writing proficiency among students (Rehan et al., 2022). The educational environment significantly impacts individuals’ comprehension of writing, expression, and technical proficiency (Graham and Rijlaarsdam, 2016). Nevertheless, writing is not solely determined by external factors; rather, it reflects an individual’s relationship with the external world and their internal self (van Manen, 2006). To effectively instruct writing, teachers must identify the cognitive patterns of students based on their emotional needs (Collins et al., 2018). At the cognitive level, attention and motivation significantly impact writing performance, indicating that the writing process necessitates deep cognitive engagement (Cleary, 1991). Consequently, exploring the cognitive mechanisms and patterns in the writing process is imperative to enhance teaching methods and individual writing abilities. Additionally, during childhood, individuals have yet to establish a distinct cognitive system; thus, their cognition is greatly influenced by human factors such as teachers and parents (Goswami and Bryant, 2007). Within the framework of school education, writing instruction represents an effective means of fostering cognitive development and enhancing writing skills among children (Graham and Perin, 2007). Graham (2019) emphasizes that to excel in writing and surpass inhibitors of effective composition, teaching must address multiple facets, including policy, school culture, and the classroom environment. In modern teaching practices, writing instruction has moved beyond the traditional confines of paper-based formats, with numerous studies examining innovative forms such as K-12 education (Murphy, 2019), computer-mediated writing (Li, 2018), dream writing (Helin, 2019), and conceptual writing (Graham, 2018). For example, Suthers and Rosen (2012) used ENA to analyze the pathways students construct scientific arguments in collaborative writing; Li et al. (2021) and Stegmann et al. (2012) used ENA to compare the emotional and reasoning connection patterns in narrative writing between high- and low-grade elementary students; Elmoazen et al. (2022) also pointed out in their systematic review that ENA has great potential in the cognitive assessment of elementary school writing. Nevertheless, writing on paper remains a significant component in Chinese educational settings. Given the need to comprehend the cognitive underpinnings of writing, this study embarked on a longitudinal investigation of the writing curriculum at X Primary School in Wuhan, China. Over six consecutive years, the study tracked and cataloged the written output of a single class. Using epistemic network analysis, the study aimed to illuminate the cognitive developmental trajectory in children’s writing. The paper delves into the interconnections between distinct writing themes, emotions, grade levels, and cognitive levels. The findings underscore the nuances of cognitive shifts in children’s writing, thereby offering practical insights for refining teaching strategies and advancing the scholarship on children’s writing trajectories.

### 1.1 Learning to write and writing education

Writing serves as a crucial communicative tool that bridges the gap between individual and collective meaning making, that is, the process in which individuals co-create knowledge and understanding through social interaction (Döbler, 2022). It offers students a powerful means of exploring, explaining, and integrating patterns of meaning, making it an invaluable epistemological tool (Prain and Hand, 2016). When teaching students the competency of writing, it is crucial to evaluate their compositions, taking into consideration not only their linguistic proficiency but also the creativity and logical coherence demonstrated in their short texts. Furthermore, students’ personal life experiences and emotional expression transformation abilities significantly contribute to the richness of their writing expression (Cheung, 2016). Other scholars have emphasized the critical role of reading in fostering writing skills, highlighting the interconnectedness between reading and writing and the need to integrate both literacies to enhance writing abilities (Graham, 2020). For example, Bereiter and Scardamalia (1987) proposed that writing is a process of knowledge transformation; Kellogg (2008) emphasized that writing involves the coordination of multiple tasks and the management of cognitive load; Flower and Hayes (1981) classic model indicates that writing is a goal-oriented problem-solving behavior. The scope of references has been expanded to provide a more comprehensive perspective.

From a developmental perspective, writing relies heavily on the utilization of basic language skills. For instance, Abbott et al. (2010) conducted a study examining students’ writing ability across grades 1 to 7 and observed that individual differences in spelling ability significantly impact their writing composition. Other research has emphasized the importance of basic writing, vocabulary accumulation, and sentence-level skills for novice writers, particularly second language learners, and young primary school children (Berninger et al., 2010; Hod et al., 2020). Beyond these foundational components, essay excellence necessitates the integration of theme, real content, emotions, reasoning, and innovation (Gilmore et al., 2019). For young writers, the mastery of writing skills demands sufficient learning time and cognitive space, making the development of large-scale, high-cognitive exercises challenging. While high-level writing skills predict the writing quality of older students, they fail to predict the writing quality of lower-grade students (Limpo et al., 2014). This highlights the importance of aligning writing instruction with children’s cognitive development stages to effectively enhance their writing proficiency. To gain insights into the factors driving writing motivation, scholars have examined the impact of teachers’ judgments, self-efficacy, and grade level on children’s and adolescents’ writing motivation and ability. They found that teachers’ judgments, self-efficacy, and grade level all positively influenced writing quality (Troia et al., 2013). To enhance writing motivation and improve writing performance, researchers have employed mind mapping-based situational games to place writers in appropriate scenarios, leveraging situational experiences to stimulate their desire for expression and guide them in gradually improving their writing abilities (Fu et al., 2019). This underscores the significance of situational experiences, themes, and motivation in composition teaching, highlighting the role of cognition—especially for young writers. Graham et al. (2017) found that cognitive variables and motivational variables significantly predict the writing quality of fourth-grade students, emphasizing the importance of cognition in writing. Raoofi et al. (2017) further discovered that students with higher writing ability tend to utilize more metacognition and cognition when mobilizing their writing strategies. In today’s global educational landscape, influenced by social and humanistic advancements, the subject matter and content of writing increasingly demand more flexible thinking and stronger cognitive abilities from writers as time progresses (Ferretti and Graham, 2019). The cultivation of writing thinking and cognition in the early stages greatly impacts students’ writing ability in middle school, highlighting the importance of stage cognitive analysis in primary school child writers’ teaching and research.

Although previous studies have explored the topic of motivation and cognitive characteristics, they lack longitudinal tracking of the developmental stages of children’s writing cognition, and most studies fail to present the dynamic relationships between cognitive elements in detail (Grammer et al., 2013). This study attempts to address this gap by combining the ENA method to reveal the structural characteristics of writing cognition as it changes with age. This addition responds to the reviewer’s criticism regarding the insufficient elaboration of research challenges and significance (Rasteiro and Limpo, 2023).

### 1.2 Epistemic network analysis

Epistemic Network Analysis (ENA) is an analytical method proposed by Professor Shaffer et al. (2009) and his team at the University of Wisconsin to quantitatively describe and characterize complex cognitive framework patterns between individuals or groups. ENA utilizes a network dynamic model to visualize the connection structure of conceptual framework in text data, characterize the correlation strength between different conceptual elements, and record the connection and change of professional cognitive elements in a specific field (Shaffer, 2016). In terms of coding methods, Csanadi et al. (2018) compared traditional coding and counting analysis and found that ENA can simulate temporal co-occurrence in discourse, making it more suitable for understanding students’ social cognitive activities. Traditional coding and counting analysis refers to the process of marking and counting the frequency of certain themes or concepts in a text, which usually fails to capture the dynamic relationships between concepts (Hamilton et al., 2013). For example, counting the number of emotional words in an essay is a typical counting analysis method (Dunsmuir and Blatchford, 2004). This term has been explicitly explained based on the reviewer’s suggestion.

This highly mathematically based analytical method compares the sum of content differences rather than network structures (Bowman et al., 2021). Finally, ENA forms a cognitive association network with “center of mass” and “node” as the core based on encoded data, visually demonstrating the connection strength and change of various cognitive elements in the conceptual framework during activities. Improving the readability of qualitative data provides great convenience for researchers to quantify and analyze complex semantic texts, and also brings a new idea for educational data mining research (Elmoazen et al., 2022).

In the field of education, ENA has the advantage of processing qualitative text data in the study of learning analysis. In specific learning practice scenarios, the text of the practice process is obtained to vertically explore the changes in learners’ professional cognition (Arastoopour Irgens et al., 2016), and the formative and summative evaluation of students’ longer written assignments is conducted (Fougt et al., 2018). ENA also provides researchers with powerful analytical ideas for evaluating and analyzing the communication texts produced in collaborative learning. Bressler et al. found that collaborative learning can effectively promote students’ scientific practice ability by asking students to play games, collecting their speech and converting it into discourse text, encoding it, and entering the ENA analysis process (Bressler et al., 2019). By encoding the language texts and actions generated in cooperation, the cognitive level differences of students at different levels can be identified from the perspective of learning evaluation, which can help them self-regulate their learning (Paquette et al., 2021). ENA can also be used to analyze cognitive differences between low-income and high-income students using online chat data (Cai et al., 2017), or to better understand complex social–emotional phenomena in learning communities (Hod et al., 2020). It can also be combined with social network analysis (SNA) to explore the joint role of cognitive skills and social skills in learning activities from the cognitive and social dimensions of learners (Gašević et al., 2018). In addition to exploring cognitive development from the perspective of students, researchers can also conduct a series of studies on professional cognitive characteristics of in-service and pre-service teachers through ENA, such as teaching resource management, professional knowledge, and identity as agents of educational change, etc. (Hu et al., 2018; Pantić et al., 2021; Wu et al., 2019). In terms of metacognition, which is more challenging to measure, some scholars have been able to analyze the differences among various elements of metacognition across different disciplines using ENA (Wu et al., 2020). ENA’s ability to process qualitative text data provides a powerful tool for exploring and understanding metacognition, which plays a crucial role in learning and knowledge acquisition. By analyzing the language and actions generated during learning activities, researchers can gain insights into students’ metacognitive processes, such as their ability to monitor their learning, evaluate their understanding, and adjust their learning strategies accordingly. This information can be used to inform teaching practices and improve students’ learning outcomes. ENA’s application in the field of metacognition is still in its early stages, but it holds promise for providing valuable insights into this important aspect of learning and development.

To sum up, ENA not only facilitates horizontal comparisons of conceptual framework elements in research activities or objects, revealing the correlation and progression of different cognitive levels from low to high but also presents a clear and powerful visual network. Consequently, ENA is deemed more suitable than other research methods for analyzing and studying cognitive elements in complex writing texts.

Therefore, the present study aims to unveil the evolution of children’s writing skills, emphasizing cognitive characteristics. Employing ENA technology to analyze written text, the research seeks to investigate the cognitive features and developmental trajectory of primary school children as they participate in the writing process over time. The study addresses the following questions:

What are the characteristics of cognitive networks in children’s writing?

What are the differences in children’s writing cognitive networks among different grades?

What are the differences in children’s writing cognitive networks between different genders?

In this study, “cognitive network” refers to the structural and dynamic co-occurrence relationships among cognitive elements—such as emotion, theme, reasoning, and creativity—within the writing process. This term is grounded in the integration of cognitive psychology and network science, and is quantifiable and visualizable through ENA (Shaffer et al., 2016; Siew and Kenett, 2019).

Additionally, theoretical and empirical evidence suggests that demographic variables such as gender and grade level significantly influence writing cognition. For instance, boys and girls often exhibit different emotional expression styles and thematic preferences in writing (Boscolo and Hidi, 2006). Moreover, cognitive capacities such as planning, organization, and reflection increase with age, leading to more complex cognitive networks in writing (Graham et al., 2015).

## 2 Materials and methods

### 2.1 Research background

The research team conducted a longitudinal study from 2012 to 2017 at X Primary School in Wuhan, Hubei Province, China. The study adopted the traditional classroom teaching model of teachers teaching and students listening, and was taught by a female Chinese teacher with more than 10 years of teaching experience. Within this model classroom, the instructor implemented writing tasks according to the syllabus, covering a variety of topics such as imagination, documentary, correspondence, etc., as specified in the guidelines from the Ministry of Education (Ministry of Education of the People's Republic of China, 2022). Based on the current teaching method of combining classroom teaching and after-class assignments in China, all the composition data used in this experiment include two parts: one is the writing task required by the teacher in the Chinese class, and the other is the homework assigned by the teacher. This study investigated the trend of cognitive changes in primary school students from grades 1 to 6, and the collection of writing data in the two scenarios can more comprehensively reflect the changes in students’ cognitive situation. The research group gathered writing data from the same students over a six-year period, spanning from grade 1 to grade 6. To ensure data integrity, samples of students with incomplete data due to factors such as transfer were excluded from the study. As a result, a total of 4,577 writing data for elementary school students were collected from 18 subjects, including 8 boys and 10 girls.

### 2.2 Theme–cognitive coding framework

According to Piaget’s constructivist theory, children between the ages of six or seven and eleven or twelve are in the stage of concrete operations. The rapid development of language skills characterizes this stage, the ability to perceive external features of things, and the emergence of certain abstract thinking (Piaget, 1962). Therefore, children in grades 5 and 6 may find themselves at the juncture between the concrete operational stage and the formal operational stage. Throughout this period, they might demonstrate not only relatively mature concrete operational abilities but also lay the foundation for the formal operational stage (Rohaeti et al., 2019, October).

The existing body of literature extensively explores the cognitive development of children in grades one through six and its implications for writing abilities. This progression is characterized by the gradual mastery of transforming external events, abstracting concepts, and transitioning to deductive reasoning (Fischer, 1980). However, there is a notable research gap concerning the nuanced interplay between specific cognitive dimensions—such as language, content, and thinking—and their individual contributions to overall writing proficiency in this developmental stage.

While Fischer (1980) provides valuable insights into the comprehensive reference for writing ability evaluation, incorporating criteria such as word use, expression ability, richness, creativity, and sincere feelings, there is limited exploration into how these cognitive abilities are distinctly reflected in specific writing texts. Additionally, the Chinese curriculum standards for compulsory education outline seven primary composition themes for primary school student (Ministry of Education of the People's Republic of China, 2022), encompassing landscape, people, things, narrative, letters, feelings after reading, and imagination. However, the literature lacks a comprehensive investigation into the direct linkage between these themes and observed cognitive development. Based on the Chinese curriculum standards for compulsory education, a coding framework for the topic of composition writing is proposed, and each dimension is described in detail in Table 1.

**Table 1:** Writing topic coding framework.

Writing Topic	Description	Example
Scenery	Depict natural or cultural scenes and express feelings.	我家住5楼,窗户外面有一棵大树, 这个树长得很高, 春天大树发芽就像穿着绿衣服, 夏天树叶长的很大, 为我们遮太阳, 秋天大树换了一件金黄色到的衣服, 冬天树叶全掉光了变成光秃秃的了。 My home is on the fifth floor. There is a big tree outside the window. This tree is very tall. In spring, the tree sprouts as if it were wearing green clothes. In summer, the leaves grow very big and shield us from the sun. In autumn, the tree changes into a golden yellow dress. In winter, all the leaves fall off and it becomes bare.
People	Depict characters and showcase their personality and qualities.	每日一句话:昨天, 我上音乐课, 突然换了一个新老师, 他的名字叫张老师, 他长得胖胖的。他上课的时候很搞笑。 A Sentence of the day: Yesterday, I had a music class and suddenly got a new teacher. His name is Mr. Zhang and he is chubby. He is very funny in class.
Object	Introduce the features of the items and express personal emotions.	爸爸妈妈花了3000元钱给我买了一张大书桌, 这张桌子是木头的颜色, 还带有一个大书架, 他的桌面是斜的只要做到背挺直, 脚放平, 头抬高这几点写起字来就会很舒服。我很喜欢它。爸爸妈妈, 谢谢你们。 My parents spent 3,000 yuan buying me a big desk. This desk is in the color of wood and comes with a large bookshelf. Its desktop is slanted. As long as you keep your back straight, your feet flat and your head raised, it will be very comfortable to write. I like it very much. Mom and Dad, thank you.
Narrative	Describe the course of events and share your insights and experiences.	今天我在语文书上面用红笔画妈妈批评我, 我不想听你的话, 我打了妈妈, 妈妈很伤心, 妈妈说每个人都必须要爱惜书。我觉得在书上画不好看, 我下次再也不在书上面瞎画了。 Today, I drew my mother’s criticism with a red pen on my Chinese textbook. I did not want to listen to you, so I hit my mother. She was very sad. She said that everyone must take good care of books. I do not think it looks good to draw in books. I will not draw randomly in books again next time.
Letter	Communicate emotions through letters in a standardized and sincere format.	亲爱的妈妈:您好!今天是我第一次写信, 我有很多话想跟你说, 我要谢谢您的陪伴, 谢谢您的关心, 妈妈我爱您, 我会回报您的!请您爱我儿子。 Dear Mom: Hello! Today is my first time writing a letter. I have a lot to say to you. I want to thank you for your company and your care. Mom, I love you and I will repay you! Please love my son.
After-reading	Record the reading experience and think about the content theme.	今天在学校老师给我们放一个教育片, 我看到有一个非洲小朋友长得又黑又瘦, 好像是走不动了, 我感觉她要饿死了, 那里是个很穷的地方, 我们要把我们不需要的东西送给他们。 Today at school, the teacher showed us an educational film. I saw an African child who was very dark and thin. It seemed that she could not walk anymore. I felt that she was going to starve to death. That place is very poor. We should give them the things we do not need.
Imagination	Use your imagination to create fictional stories or scenes.	我想要个四季房子, 到了春天小花开放了, 变得越来越香, 到了夏天小树长大了, 可以遮阴, 到秋天小河为快乐的田地歌唱, 到了冬天下雪的时候, 我可以跟小朋友一起打雪仗! I want a house with four seasons. In spring, the little flowers bloom and become more and more fragrant. In summer, the small trees grow up and can provide shade. In autumn, the small river sings for the happy field. In winter, when it snows, I can have a snowball fight with the children!

Furthermore, He et al. (2022) emphasizes the importance of essay organization, topic development, logical coherence, and language proficiency in the assessment of essay quality. While this provides a foundational understanding, the literature gap persists in elucidating how these four characteristics specifically align with the cognitive development outlined in earlier studies.

Addressing this research gap is crucial for enhancing our understanding of the relationship between cognitive development and writing proficiency in primary school students. The development of a writing cognitive coding framework, as undertaken in this study based on the aforementioned theories and studies, offers a promising avenue for bridging this gap. This framework, detailed in Table 2, systematically categorizes cognitive levels into language, content, and thinking, providing a structured approach for evaluating and comprehending the writing abilities of children in grades one through six. The data analysis in this study was mainly carried out from the two aspects of theme and cognition, and the composition data was encoded according to the coding framework of topic and cognition.

**Table 2:** Writing cognitive coding framework.

Cognitive level	Cognitive element	Description	Example
Language	Grammatical Norm	There is no grammatical error, the word meaning is appropriate, the sentence structure is complete, and the cohesion is smooth.	“我有一个非常漂亮的书包。那个书包第一层上面有一个芭比、墨镜和高跟鞋。那个芭比头发是黄色的, 项链是蓝色和黄色的, 衣服是粉色的, 衣服上还有4颗灰色的纽扣。” “I have a very nice bag. The bag has a Barbie, sunglasses, and high heels on the first layer. She has yellow hair, a blue and yellow necklace, a pink dress, and four gray buttons on her dress.”
	Lexical Accuracy	Words and punctuation use are reasonable, and there are no spelling mistakes.	“这件衣服是粉红色的, 上面有四个英语句子, 有两个英语句子是金银色的, 有两个是绿色的, 这四个英语句子中间, 还有STAR这个英文字母” “This dress is pink, with four English sentences. Two of them are gold and silver, and two are green. And in the middle of these four English sentences, there are the English letters STAR.”
Content	Episodic Memory	Incorporate real experiences from your own life, such as people and things.	“周末, 我们家来了一个2岁半的小妹妹, 它有个美丽的名字叫吴沁怡, 小名朵朵, 意思是小花朵。我觉得小妹妹非常可爱。” “At the weekend, our family received a two-and-a-half-year-old little sister, who has a beautiful name called Wu Qinyi, the nickname Duoduo, meaning small flowers. I think the baby sister is very cute.”
	Knowledge Transfer	In the composition, famous sentences, ideas, concepts, as well as historical events or people are cited, and effectively fit the context.	“华罗庚说:‘时间是由分秒积成的, 善于利用零星时间的人, 才会做出更大的成绩来。’” “Hua Luogeng said:” Time is composed of minutes and seconds. People who are good at using spare time will make greater achievements.”
Thinking	Imagination	To be inspired by real things, and to plan or describe events or ideas that are different from real life.	“长着一双透明翅膀的仙女站在了我的面前。“你是谁啊?“我奇怪地问。“我是仙女, 我带你去个地方。“仙女说。“轰”的一声, 我来到了一亿年后的地球。” “A fairy with transparent wings stood before me. “Who are you?” I asked curiously. “I am a fairy, and I will take you somewhere.” “Said the fairy. “Boom,” I came to the earth 100 million years later.”
	Logical Reasoning	In the composition, the influencing factors of an event or a phenomenon will be calculated, or the possible results will be derived from a certain reason.	“我觉得我们是可以上网的, 不过要少上网, 因为如果沉迷于上网玩游戏、聊天、必然会影响学习、浪费时间。上网对身体也有一定的危害, 会对视力有一定的影响, 还会使人缺乏体育锻炼。但是网络可以让我们获取知识的途径更便捷, 也可以更多接触外面的世界, 增强我们对社会的了解。总之, 我觉得我们要少上网。” “I think we can go online, but we should go online less, because if we are addicted to playing games and chatting online, it will inevitably affect our study and waste time.” The Internet also has a certain harm to the body, will have a certain impact on vision, but also makes people lack physical exercise. However, the Internet can make it more convenient for us to acquire knowledge, and it can also make more contact with the outside world and enhance our understanding of society. In short, I think we should use the Internet less.”

### 2.3 Data collection and processing

The study collected handwritten essay data from primary school students in grades 1 to 6, manually converted them into electronic versions, and gradually transcribed them into Excel sheets (Bardaglio et al., 2012). According to the theme and cognitive coding framework designed in this study, the writing data were encoded by “0” and “1” binary encoding. Among them, the theme includes 7 dimensions, and cognition includes 6 secondary dimensions, which are coded as “1” if the composition data reflects this element, otherwise it is “0.” After the coding, the encoded data was imported into ENA for the drawing and analysis of the cognitive network diagram, focusing on the relationship between topics and cognition, as well as the differences in the cognitive characteristics of primary school students of different grades and genders.

Cognitive network analysis can intuitively reflect the relationship between topics and cognition through the method of constructing networks, and can visualize the relationship between different cognitive dimensions of primary school students (Shaffer et al., 2009). In a cognitive network graph, there is a connection coefficient between every two points, and the larger the connection coefficient, the stronger the connection between the two nodes (Yanling et al., 2023). A cognitive network diagram consists of nodes and connections between nodes, and the differences between two networks can be compared by adding or subtracting from the cognitive network diagram (Csanadi et al., 2018).

The research team transcribed all 4,577 paper media writings into electronic texts, classified them, and encoded them according to the provided coding framework. In the coding process, two graduate students pre-coded 20% of the composition data and then conducted formal coding after three rounds of experiments. Eventually, the Kappa coefficient for the two graduate students’ writing cognition and topic coding demonstrated a high level of agreement at 0.862.

The descriptive statistics are shown in Table 3, which includes the number of texts, the average sentence length, and the number of words. All the composition texts acquired in this study were gathered without direct involvement in the writing process by the research team. The team intentionally avoided providing explicit instructional interventions to maintain control over the sample size. Variations in the number of essays across different grades are a natural occurrence. For example, in the first grade, where students had just begun their writing training, teachers primarily concentrated on instructing them in reading and expanding their vocabulary. Consequently, there were fewer assigned writing tasks, mostly consisting of short texts like diaries. Therefore, in the sample of all grades, the number of composition texts in first grade is the smallest, only 498, and the total number of words is 5,747. The first-grade students have an average sentence length of only 11.54 words, indicating initial challenges with their writing skills. In the second grade, as students became more accustomed to writing tasks, the teacher’s writing training increased significantly, resulting in a total of 1,141 essays. Students’ writing expression improved noticeably, with the average sentence length reaching 21.69 words—twice that of the first grade.

**Table 3:** Writing information for each grade.

Grade	Number of articles	word	Average sentence length
First grade	498	5,747	11.54
Second grade	1,141	25,446	21.69
Third grade	732	31,829	34.08
Fourth grade	671	33,554	41.84
Fifth grade	732	49,451	65.32
Sixth grade	803	53,170	49.83
Total	4,577	149,746	/

Coding statistics are shown in Table 4. As can be seen from cognitive coding frequency, Episodic Memory has a high coding frequency in narrative compositions, and Episodic memory has a high proportion in all compositions, accounting for 93.3%. The frequency of other coding elements has a significant difference. This shows that in primary school, writing is mostly carried out around concrete cognitive content such as narrative and memory (Zhu and Xiao, 2019). Secondly, in terms of cognitive content at the language level, the frequency of children’s correct use of grammar is higher than that of correct use of words, and the proportion of the two cognitive codes in the exercises with imagination as the theme is lower than that in the exercises with other themes. While “Knowledge Transfer” and “Imagination” have the lowest overall proportion and are very close to each other, but they both have the characteristics of a high proportion in a certain theme. For example, “Knowledge Transfer” has a total proportion of 20.4%. However, in the post-reading essays on the theme of sense, the proportion of “Imagination” accounted for 85.8%, and the overall proportion was 19.7%, but in the composition on the theme of scenery and imagination accounted for 71.4 and 82.1%, respectively. It shows that different levels of writing cognition are closely related to the relationship between themes, and different types of themes can stimulate single or multiple cognitive mobilization or connection. To further clarify the free writing text, subsequent analysis in ENA (https://www.epistemicnetwork.org/).^1^

**Table 4:** Cognitive frequency and proportion in each subject composition.

Topic	Grammar norm (c1)	Lexical accuracy (c2)	Episodic memory (c3)	Knowledge transfer (c4)	Imagination (c5)	Logical reasoning (c6)
Scenery (s1)	74(75.5%)	62(63.3%)	86(87.8%)	15(15.3%)	70(71.4%)	25(25.5%)
People (s2)	197(75.0%)	152(57.8%)	245(93.2%)	25(9.5%)	62(23.6%)	122(46.4%)
Object (s3)	282(73.2%)	213(55.3%)	346(89.8%)	54(14.0%)	174(46.0%)	133(34.5%)
Narrative (s4)	2,308(74.2%)	1835(59.0%)	3,016(97.0%)	399(12.8%)	356(11.4%)	1,440(46.3%)
Letter (s5)	38(79.2%)	27(56.3%)	39(81.2%)	8(16.7%)	10(20.8%)	26(54.2%)
After-reading (s6)	358(76.8%)	273(58.6%)	433(92.9%)	400(85.8%)	59(12.7%)	324(69.5%)
Imagination(s7)	152(73.4%)	105(50.7%)	105(50.7%)	32(15.5%)	170(82.1%)	95(45.9%)
Sum	3,409(74.5%)	2,667(58.3%)	4,270(93.3%)	933(20.4%)	901(19.7%)	2,165(47.3%)

c: Represents the number of cognitively encoded “1” in a writing topic. s: Represents the number of essays that belong to the theme. c/s: Represents the proportion of the cognition in the composition of the writing topic.

## 3 Results

### 3.1 Writing theme—epistemic network analysis

**Figure 1:**
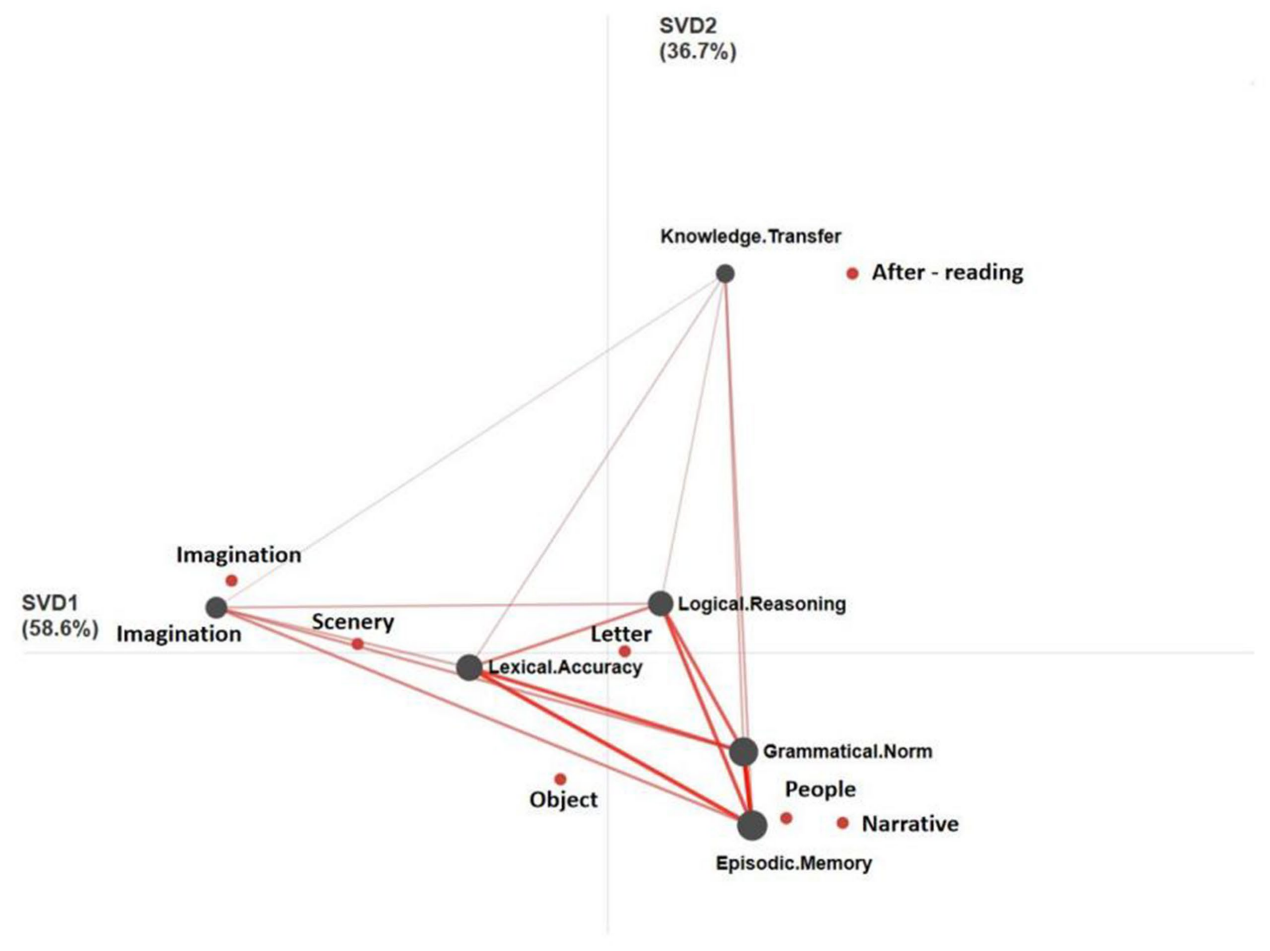
Overall topic-cognitive network relationship.

Figure 1 shows the overall ENA network diagram between the writing topic and cognition constructed with the overall centroid as the origin. The red dot corresponds to the theme of writing, and the gray dot corresponds to the cognition of writing. The spatial position of the “theme” point and the “cognition” point indicates the correlation between them. The closer the distance, the stronger the correlation. The essays on “letters” are close to the center node in the cognitive network, which indicates that the cognitive dimensions involved are relatively balanced, and there is no obvious tendency. “Perception after reading,” “imagination,” “people” and “narrative” are distributed at the edge of the cognitive network diagram, and “people” and “narrative” are between the cognitive dimensions of “Grammatical Norm” and “Episodic Memory.” “Imagination” and “perception after reading” are located in the cognitive dimensions of “Imagination” and “Knowledge Transfer” at the other two ends, respectively. “Scenery” is close to “Lexical Accuracy,” indicating that pupils pay attention to the use of words to depict scenery. The extreme bias between thematic and cognitive distribution suggests three messages. First, in the writing topics centered on “people” and “narrative,” the cognitive development of primary school students focuses on language and content; second, in the cognition level of “Knowledge Transfer,” only the theme of “feeling after reading” has an obvious connection with this cognition; third, in the higher-order cognition, The essay on the theme of “Imagination” focuses on the higher-order cognition of “Imagination” (c5), but the relationship with other levels of cognition is very weak.

In ENA, the coefficient of node connection indicates the correlation between the two, and the larger the coefficient, the stronger the correlation. Table 5 reveals the connection coefficient between cognitive nodes, and the correlation degree between language level and content level as well as content level and thinking level shows inconsistency. “Grammatical Norm-Episodic Memory” (*c* = 0.36) has a strong correlation with “Lexical Accuracy-Episodic Memory” (*c* = 0.33). The correlation between Grammatical Norm-Knowledge Transfer (*c* = 0.21) and Lexical Accuracy-Knowledge Transfer (*c* = 0.18) is relatively weak. “Episodic Memory-Imagination” (*c* = 0.26) has a strong correlation with “Episodic Memory-Logical Reasoning” (*c* = 0.31). The correlation between “Knowledge Transfer-Imagination” (*c* = 0.15) and “Knowledge Transfer-Logical Reasoning” (*c* = 0.16) is weak. “Episodic Memory” plays a great role in connecting cognition at the linguistic level with that at the mental level.

**Table 5:** Connection coefficient of cognitive nodes in the ENA network.

Connection coefficient(c)	Lexical accuracy	Episodic memory	Knowledge transfer	Imagination	Logical reasoning
Grammatical norm	0.31	0.36	0.21	0.24	0.3
Lexical accuracy		0.33	0.18	0.22	0.26
Episodic memory			0.22	0.26	0.31
Knowledge transfer				0.15	0.16
Imagination					0.21

### 3.2 Differences in writing cognition among different students

**Figure 2:**
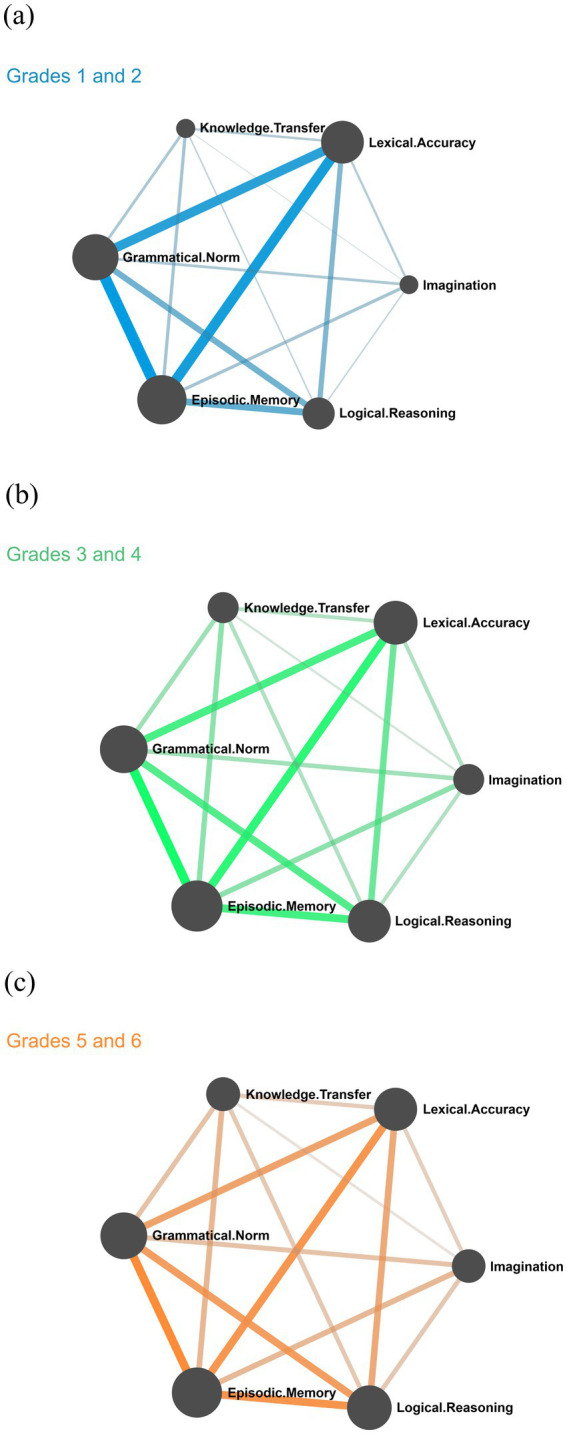
Cognitive network distribution of each grade level. **(a)** Grades 1 and 2. **(b)** Grades 3 and 4. **(c)** Grades 5 and 6.

The Chinese Language curriculum standards will be the primary school Chinese requirements by the “six three” school system to divide the arrangement, that is, the first grade level for grades one and two, the second grade level for grades three and four, and the third grade level for grades five and six. This study divides the six grades into three groups, including the first and second grades, the third and fourth grades, and the fifth and sixth grades. The distribution of grade levels in the cognitive network diagram is shown in Figure 2. Among them, blue, green, and yellow, respectively, correspond to the first and second grade, third and fourth grade, and fifth and sixth grade three sections. There are obvious differences in writing cognitive changes among different grade groups. The cognition of writing in the first and second grades is located in the first and fourth quadrants and has a strong correlation with Lexical Accuracy, Grammatical Norms, and Episodic Memory. In the cognitive network distribution, a language-content cognition triangle is formed, which highlights the primary school students learning and application of language cognition, and the extraction of memory content occupies the focus of the whole stage of writing, and the connection between language level and other cognitive levels, especially the level of thinking cognition, is weak. With the growth of grade, the center of mass gradually approaches the higher-order cognitive node. To further reveal the relationship between children’s cognitive development and grade growth, I calculated the Pearson correlation coefficient of cognitive nodes in different age groups (see Table 6), and it can be seen that with the growth of grade, cognition at the content level and thinking level becomes stronger, and different cognitive contents show significant differences among different grades. Based on the connection coefficient of cognitive nodes (see Table 7), it can be seen that with the growth of grade, the correlation degree between “Knowledge Transfer” and cognition at the thinking level as well as the correlation degree of “Imagination-Logical Reasoning” at the thinking level presents an increasing trend. It shows that children’s cognitive development tends to strengthen higher-order cognition.

**Table 6:** Cognitive-grade Pearson correlation analysis.

	Grammatical norm	Lexical accuracy	Episodic memory	Knowledge transfer	Imagination	Logical reasoning
Grade	−0.290*	−0.520**	−0.520**	0.213**	0.180**	0.324**

*p < 0.05, **p < 0.01, ***p < 0.001.

**Table 7:** The connection coefficient of the ENA network of cognitive nodes between grade groups.

Connection coefficient	Grade	Lexical accuracy	Episodic memory	Knowledge transfer	Imagination	Logical reasoning
Grammatical norm	Grades 1 and 2	0.3	0.33	0.27	0.26	0.27
	Grades 3 and 4	0.28	0.21	0.25	0.25	0.28
	Grades 5 and 6	0.27	0.3	0.25	0.25	0.28
Lexical accuracy	Grades 1 and 2		0.33	0.22	0.22	0.24
	Grades 3 and 4		0.31	0.21	0.22	0.25
	Grades 5 and 6		0.3	0.21	0.21	0.26
Episodic memory	Grades 1 and 2			0.32	0.32	0.32
	Grades 3 and 4			0.29	0.31	0.3
	Grades 5 and 6			0.29	0.29	0.3
Knowledge transfer	Grades 1 and 2				0.05	0.1
	Grades 3 and 4				0.14	0.2
	Grades 5 and 6				0.15	0.22
Imagination	Grades 1 and 2					0.1
	Grades 3 and 4					0.21
	Grades 5 and 6					0.24

### 3.3 Gender differences in writing

**Figure 3:**
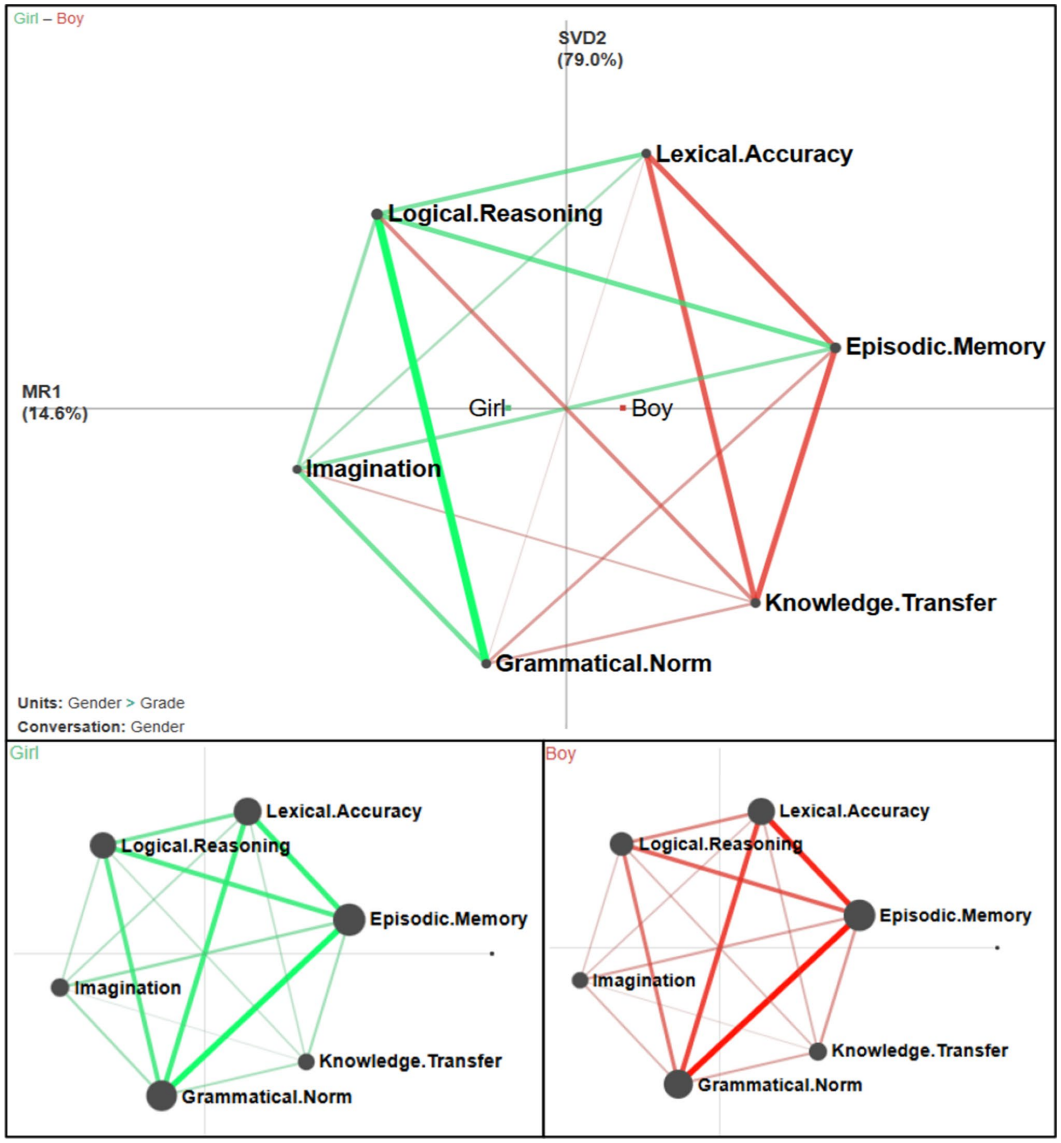
The network of male–female cognitive differences in writing. The green line represents the female cognitive weight connection, and the red line represents the male cognitive weight connection.

Figure 3 presents the male–female writing cognitive network. The right side of Figure 3 shows the respective writing cognitive networks of male and female students, where green represents boys and red represents girls. The upper side of Figure 3 shows an Overlay Plot of male and female cognitive networks. The red line indicates that the cognitive connection intensity of male students in this part is stronger than that of female students in this part, and the green line is vice versa. Combined with the connection coefficient of cognitive nodes (see Table 8), it can be seen that the cognitive relevance of male and female students is the same at the language level. Male students’ cognitive advantage in writing is concentrated in the formation of cognitive connection areas centered on “Knowledge Transfer.” Female students’ cognitive advantage in writing is concentrated in the cognitive connection area centered on “Logical Reasoning” and “Imagination.”

**Table 8:** The connection coefficient of the ENA network of cognitive nodes between different genders.

Connection coefficient	Sex	Lexical Accuracy	Episodic Memory	Knowledge Transfer	Imagination	Logical Reasoning
Grammatical norm	Boy	0.8	1	0.39	0.34	0.64
	Girl	0.8	0.95	0.36	0.4	0.73
Lexical accuracy	Boy		0.92	0.33	0.27	0.56
	Girl		0.86	0.26	0.31	0.61
Episodic memory	Boy			0.47	0.42	0.73
	Girl			0.41	0.47	0.78
Knowledge transfer	Boy				0.13	0.32
	Girl				0.1	0.25
Imagination	Boy					0.26
	Girl					0.3

## 4 Discussion

Despite the central role of writing in early education, research on the written texts of primary school students remains limited (Philippek et al., 2025; Ruffini et al., 2023). This study demonstrates that analyzing the cognitive networks embedded within students’ writing across different grade levels provides valuable theoretical insights into their cognitive development. It also introduces a novel methodological perspective for educational research by visualizing how cognitive structures evolve over time.

The findings highlight a clear relationship between writing cognition and age-related cognitive development in primary school. As students advance through the grades, their written work reflects a developmental progression—beginning with the acquisition of basic language skills, followed by increasing awareness of object characteristics, then moving to structured problem-solving, and finally to the development of logical reasoning and abstract thinking. This trajectory aligns closely with Piaget’s (2012) theory of cognitive development, which emphasizes the shift from concrete to abstract thought during childhood.

Our analysis of writing samples from grades one through six further reveals that children’s cognitive expression is strongly shaped by the themes they address. The interaction between content and cognitive depth suggests that certain topics may elicit more advanced forms of reasoning, depending on the student’s developmental stage. Moreover, the study identifies distinct cognitive patterns not only across grade levels but also between genders, highlighting the multifaceted nature of cognitive growth in writing. These findings underscore the importance of differentiated and developmentally informed writing instruction that takes into account both age and gender-related differences in cognition.

### 4.1 Writing theme—cognitive network characteristics

Overall, the cognitive differences, whether single or multiple, among subjects are evident. The distribution patterns indicate a strong correlation between various subjects and the displayed cognitive characteristics. From the perspective of the cognitive relationship of each dimension of a single subject, at the linguistic level, the compositions with the themes of “people,” “things” and “narrative” have the most obvious cognitive development in the Grammatical Norm and Lexical Accuracy. Episodic Memory at the content level and Logical Reasoning at the thinking level exhibit a strong cognitive correlation. This correlation may be attributed to their increased exposure to vocabulary and ideas associated with these topics. Writing education in primary schools often emphasizes the use of basic grammar and words (Aladrovi Slovaek and Matkovi, 2024), and the importance of “Episodic Memory “in the cognitive network diagram also reflects that most writing exercises are mainly narrative essays, requiring students to strengthen their ability to describe facts. Vita et al. (2021) posit that narratives with imagination are more dynamic than mere descriptions. This teaching method demands less creativity, and its advantage lies in the comprehensive exercise and swift enhancement of children’s personal language skills during primary school. While students can engage all three levels of cognition simultaneously in the writing of these three topics, there are evident shortcomings in knowledge transfer and imagination. To address this deficiency, composition training focusing on the themes of “sense after reading” and “imagination” is necessary. In contrast, in the theme compositions of “scene” and “letter,” the characteristics of cognitive connection shown by students are not obvious, but the overall connection is more “average,” indicating that most primary school students do not have strong experience or knowledge in the writing of these two topics, which just helps them balance and mobilize the cognition between the three levels to complete the writing of this topic.

### 4.2 Cognitive differences in writing across grades

According to the cognitive distribution characteristics of various dimensions presented by the cognitive network of different school age groups, writing cognition in primary school shows the following trends: The writing cognition of the first and second-grade students mainly focuses on the language level, which is partially connected with the content and thinking level, but the connection is weak; After the third grade, the cognitive correlation between the content level and the thinking level is gradually strengthened, and the cognitive combination tends to be integrated, from the combination of a single or a few aspects of cognition to the multidirectional connection from the language level to the thinking level. “Knowledge Transfer” at the content level and “Imagination” at the thinking level have a weak presence in the writing cognitive network throughout primary school, although the cognitive connection between the two aspects has been strengthened with the increase of grade level. However, compared with the overall cognitive change, the progress trend is still not obvious. According to the new structuralism, the cognitive ability of primary school students in childhood is relatively low (Uzundag and Aylin, 2018), and their understanding and creation of things are still in a relatively preliminary stage. The cognition of things is more dependent on the experience formed by their own experiences. Under the influence of the dual factors of teaching and immature cognitive ability, His writing will show cognitive characteristics with language and memory as the core. With the increase in grades, the Logical Reasoning ability of primary school students plays a more obvious role in cognition, which also indicates the obvious differences in cognition among age groups (Marks et al., 2023).

### 4.3 Cognitive variations in writing between genders

The difference between the genders highlights the degree of involvement and connection between boys and girls in different levels of cognition in the writing process, and there is almost no difference in the connection at the language level. While some studies have suggested that girls have an advantage in grammar and vocabulary (Henry et al., 2012), Adams and Simmons (2019) analyzed the differences in writing-related skills between students in the first and second grades of elementary school and reported that girls have no significant advantage in areas such as vocabulary. Examining cognitive aspects in writing reveals differences between boys and girls in two primary categories: content cognition and thinking cognition. Boys excel in content cognition, demonstrating proficiency in using knowledge and factual information in their writing. On the other hand, girls show strength in thinking cognition, particularly in the imaginative and logical dimensions of their writing. This analysis highlights divergent cognitive strengths between genders, emphasizing the varied approaches and skills each brings to writing abilities.

In terms of cognitive development from the content level to the thinking level, boys focus on “Knowledge Transfer” and girls focus on “Episodic Memory “, indicating that boys tend to pay attention to history, science, and other related fields, while girls tend to pay attention to personal life. This shows that even in the primary school stage where cognition is not mature, there are still distinct differences between male and female students in cognition and understanding. Male students are better at sorting out and applying rational knowledge in cognition, while female students are more interested in perceptual content, prefer to integrate elements of the real world with things in their imagination in writing, and have a stronger curiosity about things. The conclusion of the study that there are differences in the cognitive characteristics of boys and girls is consistent with previous studies (Lui et al., 2021). It makes them want to know more about how things happen. This may be related to the influence of gender stereotypes (Nanova et al., 2024). Since boys are often expected to be good at history, technology, and culture, while girls are defined as paying more attention to personal emotions and interpersonal relationships, children will be guided by such stereotypes and show corresponding expectations (Leaper, 2015).

### 4.4 Implications

This study offers a proof of concept for the refinement of writing teaching strategies. It reveals distinct cognitive tendencies in primary school students’ writing across various themes, highlighting divergent cognitive connections in language, content, and thinking aspects. Specifically, when engaging with everyday subjects or individuals, students demonstrate closer mobilization and connection at the language and content levels. In contrast, when dealing with abstract and logical concepts, the formation of a comprehensive cognitive connection system proves challenging. These findings imply the need for tailored teaching strategies that consider the distinct cognitive demands associated with different writing themes and levels of abstraction.

There are significant differences in writing cognition among students of different grades and genders, including cognitive transitions and stages, as well as perceptual or rational cognitive tendencies. Consequently, prioritizing the alignment of diverse topics is crucial in writing education, with particular emphasis on nurturing imaginative thinking abilities. Writing instruction in primary school should be developmentally appropriate and tailored to the specific needs of students at different grade levels. Targeted and personalized guidance is essential to support students’ growth as writers. For younger students, instruction can focus on expressing everyday experiences and personal narratives. At this stage, emphasizing foundational writing skills helps cultivate basic language abilities and builds confidence. In contrast, upper-grade students benefit from opportunities to explore creative and diverse writing genres. Encouraging them to experiment with new formats and storytelling techniques can foster more advanced logical reasoning and narrative structure in their writing (Martino et al., 2024).

Moreover, given the naturally vivid imagination of primary school students, it is important to nurture their capacity for innovative thinking through writing. Integrating playful and imaginative elements into writing instruction—such as storytelling games, visual prompts, or role-play scenarios—can significantly enhance students’ engagement and motivation to write (Nurkilah, 2021). By aligning writing instruction with students’ developmental stages and creative potential, educators can better support both cognitive and expressive growth throughout the primary years.

In the evolution of a new writing education model, it is essential not to blindly pursue uniform teaching methods, but rather to guide students in integrating perceptual and rational thinking. This approach aims to facilitate the holistic development of their writing expression. Changing classroom writing practice is a daunting challenge (Graham, 2019) but teachers should also make more efforts, such as making a plan to balance reading and writing education, to help students better integrate knowledge content into composition (Graham et al., 2018).

### 4.5 Limitations

Despite the valuable insights gained from this study, it is crucial to acknowledge and address several inherent limitations that impact the scope and generalizability of the findings. Firstly, the small sample size, consisting of only 18 students, may impact the representativeness of the research results, cautioning against extrapolating the findings to a broader population. Secondly, the extensive collection of written texts over a six-year period, while contributing to a rich dataset, introduces a time-consuming aspect to data collection and sorting. This delay in result feedback may hinder the timeliness of drawing conclusions, limiting the study’s immediate applicability to educational practices. The adoption of the epistemic network analysis method, though insightful, introduces subjectivity in coding, with the manual transcription and coding process being both time-consuming and labor-intensive. Lastly, the study’s focus on the Chinese writing teaching of a specific primary school in Wuhan may limit the applicability of the research results to other provinces and cities. Variations in teaching materials, methods, and writing modes across regions may limit the transferability of the coding framework and research outcomes.

## 5 Conclusion

This study employed epistemic network analysis (ENA) to examine written texts produced during different stages of Chinese writing development in classroom settings. The findings demonstrate that ENA is an effective tool for uncovering the relationship between students’ cognitive processes and subject matter, while also highlighting cognitive differences across grade levels and between genders. By mapping the developmental trajectory of writing cognition in primary school students, this study offers valuable insights for qualitative research on writing ability.

The results not only inform strategies for writing instruction but also identify targeted areas for improvement—such as strengthening post-reading and letter writing, enhancing students’ ability to apply knowledge and reason logically, and fostering a writing environment that supports both imagination and coherence, particularly for boys. From a content analysis perspective, ENA proves to be a powerful method for revealing the nuanced cognitive structures embedded in student texts, providing a strong foundation for future research design and pedagogical innovation.

By integrating ENA into the study of Chinese writing, this research captures the complex, subjective nature of cognitive development through a data-driven lens. Moving forward, it is essential to combine both qualitative and quantitative approaches to gain a more comprehensive understanding of students’ cognitive growth. This study contributes to a deeper understanding of the cognitive dimensions of writing and presents valuable directions and challenges for future inquiry in educational research.

## Data Availability

The datasets presented in this article are not readily available because this dataset is not public. Requests to access the datasets should be directed to Gyuan1027@gmail.com.

## Footnotes

^1^ https://www.epistemicnetwork.org/
